# Glioblastoma: new therapeutic strategies to address cellular and genomic complexity

**DOI:** 10.18632/oncotarget.23476

**Published:** 2017-12-20

**Authors:** Xue Cai, Michael E. Sughrue

**Affiliations:** ^1^ Department of Neurosurgery, University of Oklahoma Health Sciences Center, Oklahoma City, OK 73104, USA

**Keywords:** glioblastoma, molecular mechanisms, gene therapy, immunotherapy, CRISPR/Cas9 genome editing

## Abstract

Glioblastoma (GBM) is the most invasive and devastating primary brain tumor with a median overall survival rate about 18 months with aggressive multimodality therapy. Its unique characteristics of heterogeneity, invasion, clonal populations maintaining stem cell-like cells and recurrence, have limited responses to a variety of therapeutic approaches, and have made GBM the most difficult brain cancer to treat. A great effort and progress has been made to reveal promising molecular mechanisms to target therapeutically. Especially with the emerging of new technologies, the mechanisms underlying the pathology of GBM are becoming more clear. The purpose of this review is to summarize the current knowledge of molecular mechanisms of GBM and highlight the novel strategies and concepts for the treatment of GBM.

## INTRODUCTION

Glioblastoma (GBM) is one of the most aggressive and malignant brain tumors in humans. Because of the cell infiltration, rapid invasion, high frequency of relapse (due to a small group of stem-like cells maintained in GBM, GSCs), and very poor prognosis and survival rates, GBM is defined as grade IV glioma by WHO (World Health Organization). GBM is classified as four distinct subgroups (Proneural, Neural, Classical and Mesenchymal) [1, 2] based on different gene expression patterns, and the patients of these subtypes exhibit different genetic abnormalities and clinical characteristics, survival time, and responses to therapeutic treatment. Great efforts using multiple advanced technologies, including whole genomic sequencing, have been made to discover the molecular mechanisms underlying GBM pathology, and numerous novel discoveries have furthered our understanding of the biology of GBM in depth. The development of effective therapeutic treatments for GBM requires multidisciplinary approaches based around the known pathophysiologic mechanisms of migration, invasion and recurrence.

For many years, investigators have pursued targeted molecular therapies for GBM, such as molecular pathway inhibitors, without clinical success. We would suggest that much of this is due to the complexity of the GBM disease, which has become increasingly clear in recent years. The presence of cellular heterogeneity, which can be stunning, genetic and phenotypic heterogeneity, the remaining of GSCs, and the drug delivery issues created by the blood brain barrier (BBB), all of which makes the likelihood of a single agent treatment for GBM unlikely.

**Figure 1:**
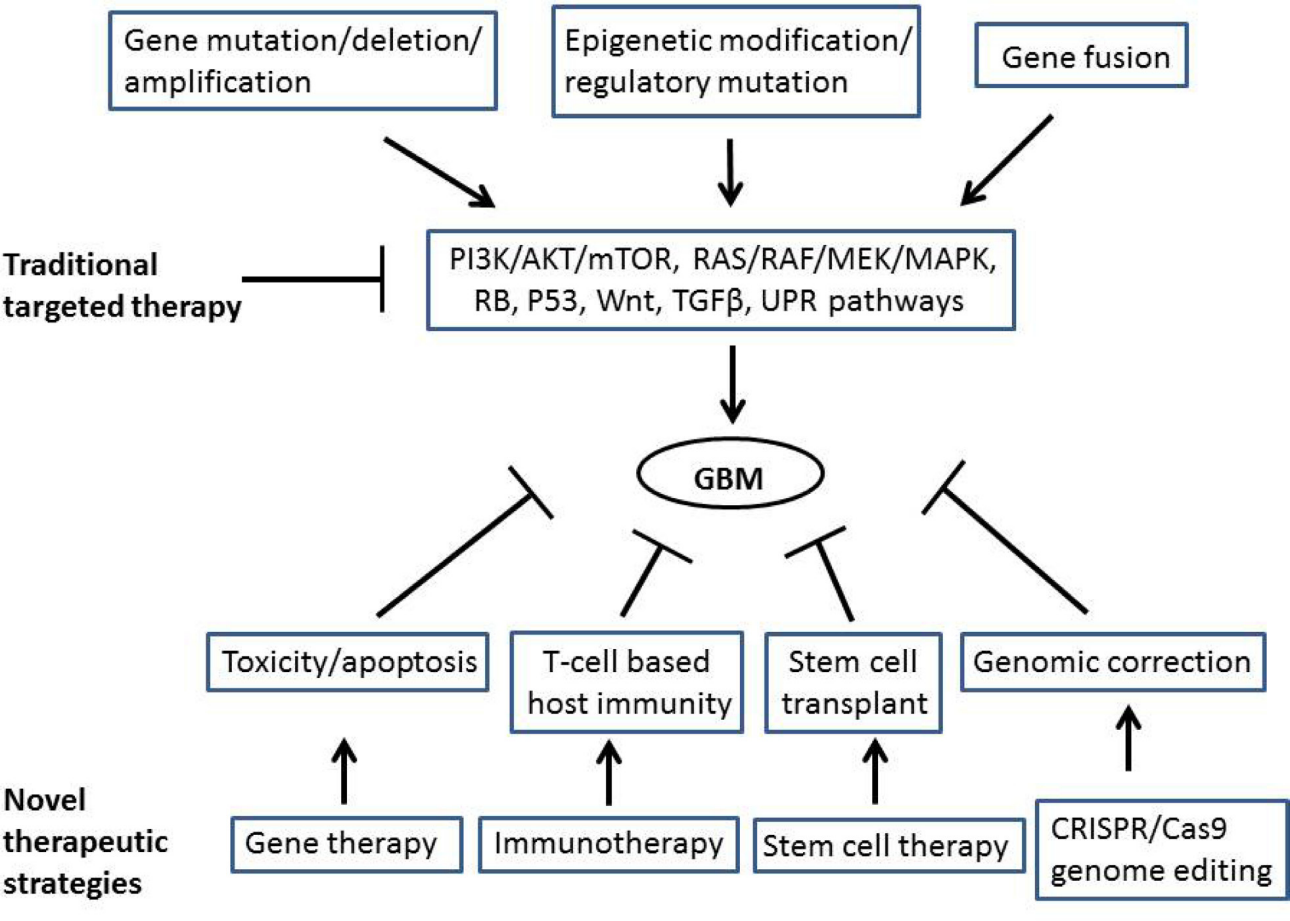
Molecular mechanisms of glioblastoma (GBM) pathologies and therapeutic strategies for GBM treatment DNA sequence alterations including gene mutation/deletion/amplification, gene fusion, and epigenetic modification as well as regulatory mutation result in activation of multiple signaling pathways, which contribute to GBM development. Traditional targeted therapies are using inhibitors targeting the signaling pathways. However, novel therapeutic strategies are signaling pathway independent. Gene therapy, immunotherapy, stem cell therapy and CRISPR/Cas9 technology aim to deliver a gene to produce toxicity to cancer cells or cause cancer cell apoptosis, enhance host immune responses, and correct the mutated genome, which consequently will reduce/cure GBM.

In this review, we profile a few key observations about driving events in GBM, and summarize facts about the extent of its complexity. We then point out some of the more promising ideas we have encountered (at least in our views), in recent years (Figure 1).

### The molecular and cellular complexity of GBM

Significant recent progress in identifying the genomic alterations of GBM by large scale somatic genomic landscaping, such as The Cancer Genome Atlas (TCGA), with other efforts, such as epigenomic, transcriptomic and proteomic analyses [3], have provided insight into the mechanisms behind tumor pathogenesis. Gene mutations, amplification, modification and rearrangement, which result in inactivation of cancer suppressor genes or activation of oncogenic genes (drivers), and subsequently activation of multiple signaling pathways, are the principal genetic causes of GBM.

As the subsequent paragraphs demonstrate, we are becoming increasingly aware that the genomic problem we face in treating these patients is a substantially complex one, making simple solutions less realistic in light of existing facts.

### Gene mutation, deletion and amplification

To date, more than 140 gene mutations have been reported in GBM (adapted data from [4, 5]). GBM patients usually have more than one gene mutated in their genome, some of them have hypermutations (untreated: average of 60 mutations/tumor; recurrent: > 500 mutated genes/tumor) [6]. It has been identified that *EGFR* (epidermal growth factor receptor), *TP53* (tumor protein p53)*, PTEN* (phosphatase and tensin homolog)*, PIK3CA* (phosphoinositide-3-kinase catalytic alpha)*, PIK3R1* (phosphoinositide-3-kinase regulatory 1)*, PDGFRA* (platelet-derived growth factor receptor α polypeptide), *ATRX* (α–thalassemia/mental retardation syndrome X-linked) [2, 3, 6, 7], *IDH1* (isocitrate dehydrogenase 1) [2, 6–8], *NF1* (neurofibromin 1) [2, 3], *RB1* (retinoblastoma 1)*, LZTR1* (leucine-zipper-like transcriptional regulator 1) [2] and *PTPN11* (tyrosine-protein phosphatase non-receptor type 11) [6] are the most frequently altered genes in GBM (primary and recurrent); and some genes are exclusively expressed in recurrent GBM including *LTBP4* (latent TGF-β-binding protein 4), *MSH6* (MutS homolog 6)*, PRDM2* (PR domain containing 2) and *IGF1R* (insulin-like growth factor 1 receptor) [6]. Comparison of gene expression levels among three gene clusters, hypermutated, mutated, and non-mutated genes, suggested that genes with hypermutations were highly expressed in recurrent tumor than non-hypermutated and non-mutated genes [6]. One remarkable phenomenon is that many gene mutations in the primary and recurrent tumors are different. Observations that mutations present in one of a patient’s two tumor samples, and not in the other, and mutations in the initial tumor are lost at recurrence suggest that divergence happened before the disease was diagnosed. Furthermore, it was observed that mutated genes, such as *EGFR*, *PDGFRA,* and *TP53*, at diagnosis can switch to a different mutation of the same gene at relapse. Additionally, specific gene mutations which are characteristic of specific GBM subtypes can switch between subtypes at recurrence, suggesting that clonal heterogeneity is a primary mechanism of treatment failure [6].

Strikingly, investigation of the factors associated with long-term patient survival (defined as survival for more than 3 years) demonstrated that amplification of *CDK4* (cyclin-dependent kinase 4) and *EGFR,* and deletion of *CDKN2A* (CDK inhibitor 2A) occurred less frequently in these patients, suggesting that these genes portend a poorer prognosis [2].

### Epigenetic modification and regulatory mutation

The term “epigenetic alterations” refers to genomic changes related to gene function and gene regulation without changes on DNA sequence [9]. Epigenetic modifications, including DNA methylation, chromatin histone modifications (ubiquitination, phosphorylation, SUMOylation and acetylation, etc.) and non-coding RNAs, play essential roles for gene regulation during cell development and differentiation. Abnormalities in epigenetic modifications have been implicated in human diseases [10] including brain tumor development and progression [11–14].

The best known example of DNA methylation in GBM is the *MGMT* promoter methylation. *MGMT* encodes O^6^-methylguanine DNA methyltransferase, and functions in DNA repair, which ultimately causes treatment failure by repairing the damage induced by alkylating agents. Low expression and methylation of *MGMT* has been shown to be significantly correlated with longer survival and better prognosis [6]. This observation further confirmed the report that *MGMT* promoter methylation reduces temozolomide (TMZ) treatment resistance [15].

Brennan and colleagues [2] reported that mutations in the promoter of *TERT* (telomerase reverse transcriptase) gene result in aberrant increase *TERT* expression and are linked to GBM pathogenesis. Point mutations of the TERT promoter are common in GBM with 83% of primary GBMs carrying the mutations. Activation of this mutated promoter in GBM enables the cells to bypass replicative senescence and overcome apoptosis for the extended lifespan of cancer cells [16]. *TERT* promoter mutation is prognostically adverse event as patients without *TERT* mutations have been observed to have a longer survival time [17].

In the past decade, the significant role of microRNAs (miRNAs, miRs) in the pathogenesis of GBM has been increasingly elucidated [18]. miRNAs belong to a class of noncoding 18–25 bp RNA sequence and have regulatory functions on a variety of cellular activities. Hundreds of miRNAs have been identified in GBM which were up regulated or down regulated, and function as oncogenes or tumor suppressor genes via mRNA degradation or translation inhibition [2, 18, 19]. They target multiple signaling pathways and each miRNA regulates numerous gene expressions [2, 18, 20]. GBM progression has often been demonstrated to result from the dysfunctional miRNA-pathway crosstalk network [2, 18, 20], and cell communication related pathways, such as focal adhesion, regulation of actin cytoskeleton, and adherens junction, were involved in the miRNA regulated pathway crosstalk module [20]. Competing endogenous RNAs (ceRNAs), which are mRNAs with competitive miRNA binding sites and are modulated by miRNAs, were predicted for four GBM signature genes (*PDGFRA*, *EGFR*, *NF1*, and *PTEN*) with each of the ceRNA correlated with different GBM subtypes: ceRNAs of *PDGFRA* and *NF1* overlapped with proneural signature genes, *EGFR* ceRNAs with classical signature genes, and *PTEN* ceRNAs with mesenchymal signatures [2].

### Gene fusion

Gene fusion is caused by the pathologic combination of two separate DNA fragments. The movement of DNA fragments from one chromosome to another chromosome or to a different site on the same chromosome can create a new gene with oncogenic properties [21]. According to the information from the fusion gene database (http://www.tumorfusions.org) (as of December 2014), over 430 gene fusions have been identified in GBM. Fusion types include in-frame or out-of-frame fusion, extended 3’ or 5’ UTR, etc., and a majority of the fusions occur at the hotspots, which are the regions frequently amplified in GBM [21].

One example of this phenomenon, FGFR3, a member of fibroblast growth factor receptor (FGFR) tyrosine kinase family, is the most frequent fusion-related gene event with multiple fusion partners. TACC3 (transforming acidic coiled-coil containing protein 3) plays a key role in maintaining microtubule organization for the spindle stability (review [22]). *FGFR3-TACC3* fusion was first described in GBM and was found in both newly diagnosed and recurrent GBM [6, 21, 23]. It was formed by intrachromosomal rearrangements of the *FGFR3* gene on chromosome 4p16, and lead to constitutive kinase activity, mitotic chromosomal segregation defects and chromosomal instability, and aneuploidy [23]. *FGFR3-TACC3* fusion resulted in the loss of miR99a, promoted cell proliferation and tumor progression in GBM cultures and xenograft mice, co-existing with amplification of *EGFR*, *PDGFR*, or *MET* genes [24]. The tyrosine kinase domain of FGFR and the highly reserved C-terminal coiled-coil domain of TACC are necessary for the oncogenic function of the fusion gene. *In vitro* assessment revealed that this fusion protein with breakpoint between exon 18 of *FGFR3* to exon 11 of *TACC3* caused increased total phosphorylation and tyrosine phosphorylation compared to the action of FGFR3 alone. Fractionation analysis demonstrated that presence of the TACC3 domain leads to FGFR3 to be located to the nucleus, cellular transformation, strong elevation of MAPK phosphorylation, and IL-3 (interleukin 3) independent proliferation [25]. Wang et al. [6] reported in-frame gene fusions involving *MGMT* gene with other genes. In addition, a novel class of gene fusion that involves the 5’ partner gene fusion with non-coding RNA genes, producing C-terminal truncation and non-coding RNA expression, was also reported [21].

Although only 1.2–8.3% GBM carry FGFR3-TACC3 fusion proteins based on the reported data [22, 24], the clinical relevance is significant. It has been reported that treatment of athymic murine models with xenografts of human glioma stem cells GIC-1123 (which carry the *FGFR3-TACC3* gene fusion) with FGFR inhibitor (JNJ-42756493) lead to marked growth inhibition and tumor regression after 2 weeks treatment [26]. Furthermore, a phase I trial (NCT01962532) using JNJ-42756493 to treat two patients with recurrent GBM harboring FGFR3-TACC3 fusions resulted in clinical improvements: one in disease stability; another has shown reduction of tumor size. Clinical phase II trial (NCT01975701) targeting FGFR1-TACC1 and FGFR3-TACC3 fusions and/or activating mutation in FGFR1, 2, or 3 by anti-tumor drug BGJ398 has been completed in December, 2015 for assessment of overall survival, anti-tumor activity, and safety and tolerability.

### Other mechanisms implicated in GBM pathogenesis

Cdc42 (cell division cycle 42) is one of the three best characterized Rho-GTPase members. It controls and regulates broad cellular activities [27] including GBM cell polarity and migration via specifying localization of filopodia [28]. Doxycycline-inducible overexpression of Cdc42 resulted in significant migration and invasion of U87MG and U251MG cells, and decreased survival of U87MG and U251MG xenograft mice. In contrast, inactivation of Cdc42 prolonged survival of both mouse models. Moreover, knockdown of CDC42 binding partner, IQGAP1 (IQ-domain GTPase-activating protein 1) also decreased cell migration, invasion and proliferation in Cdc42 overexpressed U251MG cells [28]. Interestingly, expression of Cdc42 RNA was significantly higher in the proneural and neural GBM subgroups [28].

EMR3 (EGF module-containing, mucin-like hormone receptor 3) is a G-protein coupled receptor (GPCR) with unknown ligand and cellular function. It can generate different protein isoforms through alternative splicing. The isolated two isoforms (cell surface protein and soluble protein) were identified containing two EGF-like domains. EMR3 is highly expressed in neutrophils, monocytes and macrophages, the ligand of EMR3 soluble form is located in the surface of monocyte-derived macrophages and active neutrophils, indicating its pivotal role during immune and inflammatory responses [29]. *In vitro* analysis of EMR3 function in multiple GBM cell lines demonstrated that EMR3 plays an important role in GBM migration and invasion, but has no effect on cell proliferation [30].

GRK5 (G protein-coupled receptor kinase 5) activates G protein-coupled receptors (GPCRs) by phosphorylation and GPCR desensitization. GRK5 has a diversity tissue distribution and subcellular localization; it regulates their intrinsic kinase activities. Expression of GRK5 is highly correlated with GBM aggression. The level of GRK5 is elevated in GSCs than the differentiated GBM cells; Knocking down of GRK5 decreased the proliferation rate of GSCs [31].

Octamer binding transcription factors (OCTs) control early stages of developmental regulation. OCT7, SOX2, SALL2 and OLIG2 were demonstrated to be core transcription factors required for GBM reprogramming and transition of differentiated GBM to GSCs [32]. High expression level of OCT4 contributed to chemo-resistance [32].

### pathways involved in GBM pathogenesis

**Figure 2:**
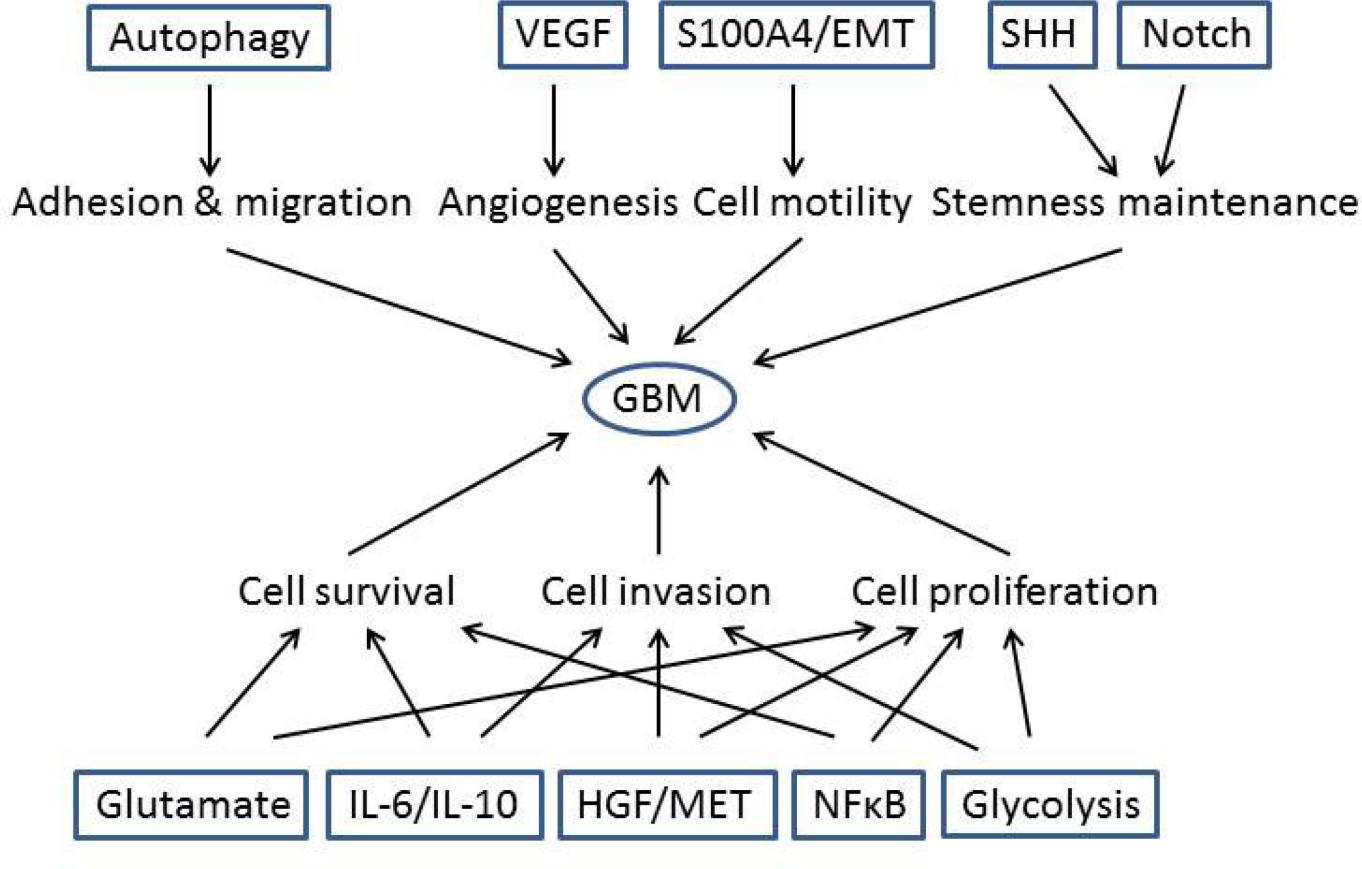
More signaling pathways have been identified to contribute to GBM development Representative additional signaling pathways were identified and regulate cell proliferation, adhesion and migration, invasion, angiogenesis, cell survival and stemness maintenance.

Three well-known core signaling pathways have been identified which are at the center of GBM pathogenesis, and which many causative GBM mutations ultimately alter. They are RTK/Ras/PI3K, Rb and p53 pathways [3]. Extensive investigation involving large numbers of GBM patient samples identified more signaling pathways and effectors [2, 20, 33–37] involved with GBM development, and demonstrated that different subtypes of GBM may involve different signaling pathway activation [2] (Figure 2).

PI3K/AKT/mTOR pathway is a well-known signaling pathway involving tumorigenesis, development, migration, invasion and relapse of GBM. Activation of the PI3K pathway is very common in GBM [3] due to amplification or mutation in EGFR or other receptor tyrosine kinases, such as *PI3K*, *PIK3CA*, *PTEN* and *NF1* [2, 3]. Targeted therapy of GBM using inhibitors of EGFR and components of PI3K/AKT/mTOR pathway or combining with other agents has been investigated, but is still in preclinical or clinical trial stages (review [38, 39]).

The RAS pathway is activated in almost all cases of GBM and is required for maintenance of KRas and Akt–induced tumors in mouse models of GBM [40]. Newborn Nestin-TVA mice were co-infected with viral vectors of KRas, *Tet-off* and TRE-Akt (TRE is a tet-responsive element), and the delivered *Akt* expression was controlled by the Tet (tetracycline) system; tumor formation began to be seen at three weeks of age; suppression of *Akt* expression with an AKT inhibitor (doxycycline) significantly decreased cell proliferation, regressed tumor size, and extended mice survival, further revealed that tumor progression and maintenance required AKT expression [41]. RAS activates the MAPK pathway (RAS/RAF/MEK/ERK). A phase II clinical trial (NCT00730262) with recurrent GBM to evaluate the efficacy of the inhibitor of Ras/MAPK signaling pathway (TLN-4601) was initiated, and the data indicated that the targeted drug was safe and well tolerated; however, this study was terminated after treatment for 4 cycles due after failing to demonstrate a clinical benefit. This is despite data in animal models that TLN-4601 is able to cross the BBB, accumulates in the tumors, and slows tumor progression [42].

Wnt signaling was first identified in cancer, and is a common signaling pathway which has been shown to regulate a variety of cellular activities including embryogenesis, cell proliferation, migration, and differentiation. Dysregulation of this pathway has been reported in multiple diseases [43, 44]. Wnt pathway is an important molecular mechanism controlling GBM (and GSCs) maintenance, proliferation and invasion. The contribution of the Wnt pathway in GBM has been reviewed [45]. Recently, Yang and colleagues demonstrated that, in GBM (NSSU2) and GSCs (U87s and SU-2) cell lines, lincRNAs (long intergenic non-coding RNAs) and miR-146b-5p were down regulated and HuR (Hu antigen R) and β-catenin were up regulated. LincRNAs negatively regulate Wnt/β-catenin signaling. HuR is activated through PI3K/AKT/mTOR pathway, its role in post-transcriptional regulation occurs via miRNAs; increased expression of HuR resulted from reduced level of miR-146b-5p. Therefore, overexpression of miR-146b-5p suppresses β-catenin through targeting HuR/lincRNA-p21 pathway. Furthermore, overexpression of miR-146b-5p reduced GSCs viability, arrested GSCs in G0/G1 phase, increased apoptosis of GSCs, reduced GSCs neurosphere formation, and promoted GSCs differentiation and radio-sensitivity. Moreover, *in vivo* analysis of miR-146b-5p effectiveness demonstrated that overexpression of miR-146b-5p decreased GSCs incidence and increased survival of mice bearing tumors [46].

TGFβ (transforming growth factor beta) signaling is associated with regulation of cell proliferation, differentiation and apoptosis [47]. Its activation and expression is correlated with cancer invasion, migration, progression and poor prognosis [47–49]. TGFβ is a positive regulator of angiogenesis, it promotes generation of immunosuppressive regulatory cells and increases macrophages capacity to produce immunosuppressive cytokine IL-10, which has been proven to act on imped anti-tumor immune responses in the microenvironment [50]. TGFβ also plays an essential role in maintenance of GSCs by regulating *Sox4* to induce *Sox2* (a stemness gene) expression [47, 48]. Besides the canonical TGFβ/Smad pathway, TGFβ directly activates several MAP kinases and corresponding MAPK signaling pathways [47]. *In vitro* experiments and clinical evidence indicate that high expression of *LTBP4* promotes TGFβ signaling pathway [6]. Also overexpression of MAP kinase-interacting kinase 1 (MNK1) occurs in GBM patient samples and GBM cell lines, knockdown or inhibition of MNK1 expression resulted in decreased TGFβ-induced Smad2 phosphorylation and cell proliferation and motility [51]. Several drugs targeting TGFβ signaling pathway have been proven effective for anti-tumor treatment in patients [48, 50].

Increasing evidence is recognizing that ER (endoplasmic reticulum) stress and subsequent UPR (unfolded protein response) activation are involved in cancer cell proliferation, migration, and differentiation, as well as metastasis (review [52, 53]). Stressful environmental conditions in cancers such as oncogene expression and aneuploidy, rapid cell division and growth, which resulting in hypoxia, nutrition deprivation, low PH etc., will trigger ER stress and activation of UPR signaling pathways by which the cancer cells utilize for survival under severe physiological and cellular conditions [52]. It has been documented that significant elevated GRP78 level (glucose-regulated protein 78 kDa) [54, 55] and increased UPR activities [55] were detected in GBM patients, cell lines [54, 55], and tumor xenograft mice [55]. Furthermore, increased expression of ER chaperones, UPR targeting genes and metabolic enzymes (glycolysis and lipogenesis) are correlated with poor prognosis of GBM patients [55]. Indeed, targeting ER stress and related components is emerging as a novel therapeutic strategy for GBM treatment. Reagents aiming to induce ER stress and activate the UPR have been shown to be effective (in combination with or without TMZ) in inducing apoptosis of GBM cells [53]. Inhibition of the activities of ER stress chaperones (GRP78 or prolyl 4-hydroxylase beta polypeptide, P4HB) has slowed cell growth, activated expression of CHOP and caspase 7 in TMZ treated cells, and enhanced GBM sensitize to TMZ treatment *in vitro* [53, 54]. Clinical phase I trial employing an ER stress-inducing cytotoxicity drug combine with TMZ and radiotherapy, and several clinical phase II trials using drugs targeting ER stress-relevant events have been conducted, and some of them produced therapeutic benefit (review [53]).

### Treatment paradigm for GBM

Conventional magnetic resonance imaging (cMRI) has been widely used for assessing the efficacy of therapeutic treatment of GBM, but its utilization was limited by only providing structural information of GBM location and disrupted/undisrupted BBB. Over the past decade, advanced imaging techniques, such as perfusion weighted imaging (PWI) for microvascular dynamics (which increases in the cerebral blood volume (CBV) in the tumor progression), diffusion weighted imaging (DWI) for water molecule diffusion (which decreases in tumor progression), are routinely used to more accurately distinguish pseudoprogression (imaging changes related with treatments, such as increase in tumor volume, oedema and enhancement) from true tumor progression, and accurately determine patient’s early-stage response to therapies, thereby contribute to aiding clinical decision [56–58]. Multiparametric imaging approach combining multiple modalities including MR spectroscopy (to examine the distribution of chemical metabolites), DWI and PWI [56, 57], or employment of other novel MRI techniques [57] is emerging in recent several years, and has shown to improve the diagnostic accuracy than single imaging to avoid over/under assessment of treatment responses.

Presently the standard-of-care treatment for GBM is maximal safe surgical resection, followed by fractionated radiotherapy plus chemotherapy with TMZ [59, 60]. Surgery alone cannot cure GBM, but we would argue that the large body of data supporting improved survival with improved extent of resection [61] and common sense says that good cytoreductive is essential for any therapeutic agent to have a reasonable chance to work. The above discussion should make it abundantly clear that unlike the rare cancer which is unimutational and homogeneous (lending to single agent treatment), that GBM is anything but unimutational and homogeneous, but instead is really several parallel diseases co-existing in the same patient. One likely explanation for previous treatment failures is the invasive infiltration represents the nature of the GBM, as in about a third of cases the tumor recurs at a distant from the primary tumor location, and the secondary tumor is often radiation and chemotherapy resistant.

The development of effective therapies for GBM has been slowed by the complex nature of this tumor which involves multiple gene mutations, gene fusions, amplification and modifications; phenotypically constant changing during tumor progression, and the genetic background heterogeneity, as well as the involvement of multiple signaling pathways, which are co-existing and cross-talking in GBM; suppression of one pathway might be insufficient to inhibit the activation of other pathways (review [62–64]). Another obstacle for the therapeutic effectiveness of GBM treatment is that the BBB blocks the passage of therapeutic drugs, including small molecules, to the targets [64, 65]. And the third, the existence of GSCs [66–68], which demonstrate the capacity of self-renewal, differentiation, and initiation of secondary tumors, is a major cause of resistance of targeted tumor therapy [62, 66].

The BBB is the major structural obstacle for drug delivery. Many strategies have been investigated for effective drug delivery by crossing the BBB, including breakage of the BBB, modification of the drug to be more lipophilic, bypassing the BBB (intraventricular/intrathecal delivery), and convention-enhanced diffusion, nanoparticles and liposomes as drug carriers, etc., [69, 70]. Among these, nanoparticle-based drug delivery system by which therapeutic molecules were loaded in the designed nanoparticles and specifically delivered to the tumor tissues has been attracted lot more attention, and shown promising in GBM treatment [69]. A variety of nanoparticles with various formulation and particle sizes were widely studied and have shown that they can protect drug degradation, control drug release for a sustained period of time, and reduce toxic side effects [69, 70], although safety concern is still needed for further investigation.

A variety of treatment strategies have been attempted with the goal of preventing, inhibiting and regressing the disease development. Treatment strategies targeting signaling pathways, transcriptional factors, and inhibitors of epigenetic factors and chaperone proteins, etc., have reached certain degree of benefit, however, infiltrative growth, migration, and multiple lesions, recurrence and stem cell-like characteristics of GBM have limited the success of single agent strategies (review [64, 71, 72]). We would argue that the nature of GBM forces us to look at multi-pathway strategies, or mutation independent strategies for GBM, including personalized targeted therapies, gene therapy, and immunotherapy.

The remainder of this review is dedicated to highlighting some of these strategies.

### Gene therapy

Gene therapy has the distinct advantage that cell death could potentially be pathways independent, which gets around the issues of clonal heterogeneity and pathway diversity which have plagued molecularly targets monotherapies. Several strategies have been employed including suicide genes which encode enzymes for converting a prodrug into an active cytotoxic compound; immunomodulatory genes to enhance immune response for antitumor; tumor-suppressor genes; and oncolytic virotherapy using viruses which are selectively to target and to induce lysis of tumor cells (review [73]). These approaches can work alone or in combination with other approaches to achieve the maximum effectiveness for tumor subtraction. Gene delivery was mediated with various carriers including viruses, stem cells (tumor-tropic neural stem cells (NSCs) and mesenchymal stem cells) and nanoparticles. Many preclinical and clinical trials employing these gene carriers and therapeutic strategies have been completed or ongoing (review [73]).

*Emx2* (empty spiracles homeobox 2) is a transcription factor bearing multiple functions in neuron development, progression and survival, its expression was undetectable in GBM patient derived cultures [74]. Overexpression of *Emx2* in U87MG and T98G cell lines and five GBM cultures from patients, in which *Emx2* was selectively activated in only the tumor cells without effect the health cells and was TetON controlled by doxycycline, caused the expansion of the cells arrested for proliferation, significant alteration on the expression of genes related with mitogenic and RTK signaling, and the genes controlling early G1 checkpoint in five GBM patient cultures, and caused the cultures collapsed within 7–8 days after treatment. By restricting *Emx2* overexpression (*Emx2* overexpression is highly toxic to neurons) in the tumor cells in xenograft mice, the antioncogenic activity of *Emx2* was evidence [74].

There are many candidate viruses capable for oncolytic virus therapy which have been proposed or employed in malignant brain tumors [75]. More recently, researchers from Duke University developed a breakthrough new immunotherapy approach, which is now in an ongoing clinical phase I trial (NCT01491893), for the treatment of recurrent GBM patients (with a karnofsky performance score ≥ 70%) using oncolytic PVSRIPO viruses, in which a single dose of 3 ml (5 × 10^7^ TCID50 tissue culture infectious dose) was directly delivered inside the tumor to destroy the cells, and the infection stimulates the host immune system to destroy other tumor cells. Safe and appreciable efficacy of the treatment was observed, and the overall survival rate was extended in half of the treated patients [76]. PVSRIPO is a genetically engineered poliovirus, it is recombinant for PV (Sabin)-Rhinovirus IRES PV Open reading frame and non-pathogenic poliovirus:rhinovirus chimera. The critical pathogenesis determinant, IRES (internal ribosomal entry site), in the genome of the living attenuated poliovirus serotype 1 vaccine was replaced with its counterpart from human rhinovirus type 2. PVSRIPO naturally infects cancer cells due to the presence of poliovirus receptor (CD155/Necl-5) in most tumor cells (review [77–79]).

Researchers from MD Anderson Cancer Center employed a modified adenovirus Delta-24-RGD (Arg-Gly-Asp motif) to treat recurrent GBM patients, in which RGD binds to integrins (the surface receptor of cancer cells) for viral internalization. This virus contains a deletion of eight amino acids in the region for binding the Rb protein and is highly expressed in cancer cells but prevents its replication in normal cells. Preclinical studies demonstrated its antitumor activity and enhanced immunity of the host [80, 81]. A phase I trial in 25 patients for assessment of toxicity showed that the highest dose (3 × 10^10^ viral particles) was well tolerated; tumors in three patients were completely disappeared and in one patient was regressed; 10–10,000 fold increased cytokine IL-12p70 in serum was detected in these patients [82]. The patients lived for more than 3 years, and clinical trials of Delta-24-RGD combining with IFNγ or PD-1 inhibitor are planned (quote from Oncolog, MD Anderson’s report to physicians, May 2016, vol 61, No.5). Recently, the same group reported the antiglioma activity in immunocompetent C57BL/6 mice of Delta-24-RGDOX expressing the immune costimulatory OX40 ligand, and synergistic inhibition of gliomas and significantly increased survival in mice was shown by intratumoral injection of Delta-24-RGDOX and an anti-PD-L1 antibody [83].

One known mechanism critical to GBM growth is angiogenesis. An increase of new endothelial blood vessels is necessary to supply nutrient for tumor growth. Targeting angiogenesis has provided us one of the four FDA approved strategies for treatment of GBM. VB-111 is an antiangiogenic drug in which a non-replicating adenovirus (Ad-5, El-deleted) containing a proapoptotic human Fas-chimera transgene (Fas and TNF receptor 1) was directed by a modified murine pre-proendothelin promoter (PPE-1–3x). VB-111 specifically targets endothelial cells within the tumor vasculature to induce apoptosis of these vessels. Preclinical study demonstrated that single intravenous dose sufficiently inhibited tumor growth within four weeks of treatment, significantly decreased microvessel density, and extended survival of U87MG xenograft rats and U251 xenograft mice [84]. A clinical phase II trial (NCT01260506) evaluating the safety, tolerability and efficacy of VB-111 in 62 recurrent GBM patients is ongoing and a phase III trial with more than 50 patients enrolled has been initiated.

IFNβ (interferon beta) is a secreted cytokine and has antiviral immune modulatory, antitumor, and antiangiogenic properties [85]. Systemic delivery of human IFNβ gene via an AAV vector has resulted in intravascular infusion, widespread gene expression and distribution in astrocytes and endothelial cells in GBM8 xenograft mice (a model of invasive GBM). Furthermore, prevention of tumor growth and complete regression of the existing tumor in a dose-dependent manner were achieved. Finally, this approach significantly improved survival rate [85].

### Immunotherapy

Immunotherapy provides the promise of a sustained antitumor immunity which is pathway independent and which has the potential for antigen expansion to further the immune response. This promise has been tempered by the realization that GBM is not a very immunogenic tumor, compared to tumors such as melanoma.

GBM exhibits significantly higher perivascular cytotoxic T cell infiltration, and perivascular and intratumoral natural killer cells and macrophages [86]. It has been demonstrated that intermediate or extensive CD8+ T cell infiltrates are associated with long-term survival in GBM patients than rare or focal T-cell infiltrates [87]. These results indicated the clinical potential of T-cell based immunotherapy. Recently, immunotherapy appears as a novel promising approach for cancer treatment and brings a new hope for GBM patients (review [88, 89]). In the microenvironment of GBM, hypoxia triggers the activation of immunosuppressive pathway, polarization of tumor associated macrophages, and overexpression of immune checkpoint genes, *PD-L1* (programmed death-ligand 1) and *CTLA-4* (cytotoxic T-lymphocyte-associated protein 4) [88]. Peptide vaccines, such as mutated form of EGFR protein (EGFRvIII), or dendritic cell vaccines as well as immune checkpoint inhibitors, anti-PD-1 and anti-CTLA-4, have been studied in clinical phase II/III trials and demonstrated improved overall survival for the patients (review [71, 89, 90]) (also see update information from National Cancer Institute http://www.cancer.gov/types/brain/research/immunotherapy-glioblastoma).

CARs (chimeric antigen receptors) T cells are synthesized molecules and modified to express receptors specific for certain types of cancers, such as patients with the EGFRvIII mutation. Preclinical experiments testing the effectiveness of CAR T cells in xenogeneic subcutaneous and orthotopic mouse models of human EGFRvIII+ GBM showed that GBM CAR T cells inhibited GBM growth, and regressed an even deeper tumor in combination with TMZ [91]. This resulted in the starting of a clinical phase I trial study (NCT02209376). Two more clinical trials using CAR T cells, one target HER2 antigen (NCT01109095), another one also targeting EGFRvIII using different vector types and CAR design (NCT01454596), to treat GBM are ongoing.

### Stem cell therapy

In one approach, embryo stem cells (ESCs)-derived astrocytes conditionally expressing TNF-related apoptosis-inducing ligand (TRAIL), controlled by a tet-on system and activated by doxycycline, were injected into human A172 cell subcutaneous xenograft mice. A significant reduction of tumor volume was seen 48 h after a single or two injections, and a 40% reduction was reached at 7 days after long-term treatment. Death receptor (DR4) expression was significantly increased in the injected mice, and apoptosis and necrosis occurred in the tumor cells [92].

Transdifferentiation (TD)-derived induced neural stem cells (iNSCs) are found to express nestin and Sox2 (markers of neural stem/progenitor cells), and differentiate into astrocytes, neurons and oligodendrocytes. iNSCs showed no cancerous teratoma formation as seen in ESCs or iPSCs (induced pluripotent stem cells)-derived NSCs ([93], review [73]), but have tumoritropic properties and selectively migrate to human GBM cells *in vitro* and *in vivo*, even when the iNSCs were implanted in the contralateral hemisphere of the brain. The engineered iNSCs expressing a secreted variant of TNF-α-related apoptosis-inducing ligand (TRAIL; iNSC-sTR) released the similar levels of the TRAIL protein to those by wild type NSCs. *In vitro* analysis demonstrated that the iNSC-sTR decreased the viability of U87 and LN18 GBM cells by more than 87% through up regulation of caspase 3/7. *In vivo* assessment indicated that iNSC-sTR treatment significantly decreased GBM volume 123 fold by day 28 in U87 GBM xenografts and greatly increased the survival time. In addition, delivery of TRAIL attenuated the progression of invasive and diffused tumors in patient-derived GBM8 xenograft mice by 18.3 fold by day 33 after treatment. iNSC-sTR also reduced the cell viability in other three more patient-derived GBM cell lines 24h after treatment. Finally, reductions of tumor volumes were seen in the xenograft mice with these cells [93].

## CRISPR/CAS9 FOR GENOMIC EDITING THERAPY

**Figure 3:**
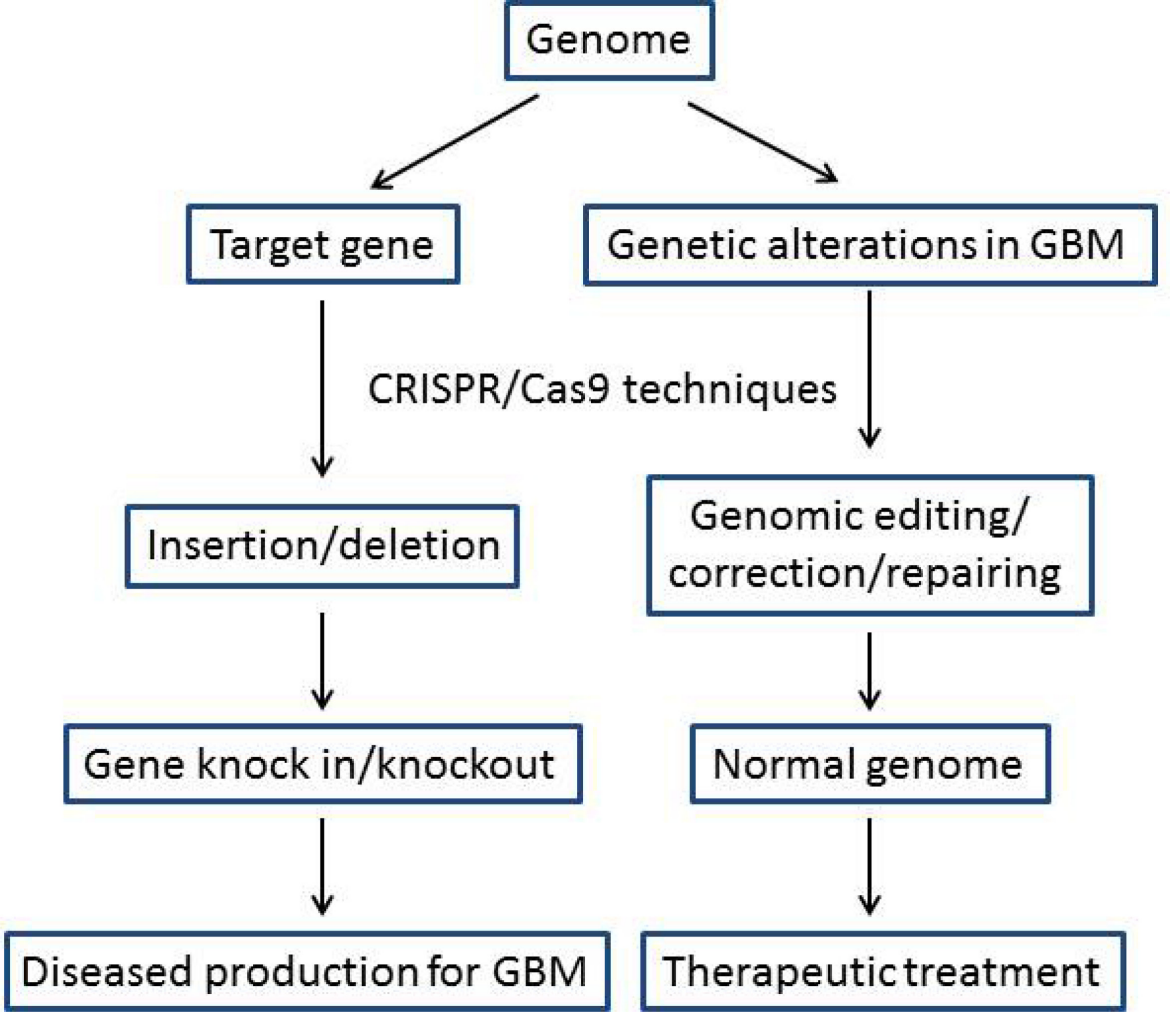
Application of CRISPR/Cas9 for pathological studies and therapeutic treatment of GBM CRISPR/Cas9 is increasingly used for generation of accurate diseased animal models to discover the real cause of the diseases by insertion or deletion to produce gene knock in and knockout. CRISPR/Cas9 is more popularly used for precise editing, correction and repairing of the alterations in the genome to therapeutically treat GBM.

CRISPR/Cas9 (clustered regularly interspaced short palindromic repeats/CRISPR associate protein 9) is a novel and attractive technique. The function of this system relies on endonuclease Cas9 to find and cut the target DNA directed by the “single-guide” RNA (sgRNA). The discovery and application of the CRISPR/Cas9 system has brought molecular biological research to a new level, and its ability for targeted and accurate genome editing, correction and repairing suggests tremendous potential for the treatment of a wide spectrum of inherited diseases [5, 94, 95]. CRISPR/Cas9 has been increasingly used for genomic silencing, and knock in and knockout for targeted gene mutation to generate more accurate diseased animal models and thereby to discover the real cause for the diseases [94, 96, 97] (Figure 3), such as CRISPR/Cas9-mediated somatic deletion of a single *Ptch1* locus or deletion of *TP53*, *Pten* and *NF1* simultaneously resulted in the development of medulloblastoma and GBM in the mouse brain [98]. Numerous successful preclinical applications of CRISPR/Cas9 technique for the treatment of various inherited diseases have been reported recently [97] (Figure 3). For example, the rd1 mouse is a model for human ocular disease (retinitis pigmentosa), it exhibits rapid and early loss of retinal function. Using CRISPR/Cas9 genomic editing technique, researchers from the University of Columbia successfully rescued the retinal structure and function in Y347X-repaired *rd1* mice [99]. In another report, the mutation of FAH causes HTI (hereditary tyrosinemia type I), which exhibits accumulation of toxic metabolites in hepatocytes and severe liver damage. CRISPR/Cas9 mediated *Fah* locus editing with co-transfection of individual and specific sgRNAs targeting *Fah* resulted in liver functional and phenotypic rescue in the mutant *Fah* mice [100]. Using viral vectors, several recent reports demonstrated the feasibility of application of this technique for *in vivo* gene delivery and corrections, and that lead to phenotypic rescue in diseased mouse models (review [101]).

Certainly this is exciting technology with potential application for cancer therapy. However, the challenge of applying this technique to a disease like GBM should be obvious from the discussion above. There are numerous cell clones, often with multiple mutations in multiple pathways per clone, and thus it is unclear which are the driver mutations to target. Finally, the elephant in the room is drug delivery, as GBM hides behind the BBB which excludes many agents. The feasibility of this approach in GBM is presently unknown, however some preliminary work in other cancers with this strategy suggests it is worth study. For example, Gebler and colleagues [4] reported that CRISPR/Cas9 system is sensitive enough to distinguish single base pair alteration and selectively cleavage cancer mutant genes. In addition, when several specific sgRNAs were simultaneously supplement with CRISPR/Cas9 system, multiple cancer gene mutations can be targeted at the same time. These exciting discoveries indicate that the CRISPR/Cas9 is a promising approach and will not only be effective for a variety of inherited disease treatment, but also pave the way for other diseases like cancer based in DNA alteration.

## CONCLUSIONS

GBM treatment has proved elusive, despite decades of research. Although many novel therapeutic strategies have been tried, the complexity of this disease has thwarted these efforts, and this continues to push us into newer strategies which deal with many of the challenges posed by this disease. Certainly the ideal strategy is unclear; however we are excited about strategies which have the potential to be pathway independent, and to get around the heterogeneity of this problem. Examples like gene therapy and immunotherapy and the newly emerged CRISPR/Cas9 genome editing technology, offer the possibility of addressing this complexity, and we would suggest, represent the most promising ideas currently in exploration for this disease.

## Abbreviations

ATRX: α–thalassemia/mental retardation syndrome X-linked
BBB: blood brain barrier
CARs: chimeric antigen receptors
CBV: cerebral blood volume
Cdc42: cell division cycle 42
CDK4: cyclin-dependent kinase 4
CDKN2A: CDK inhibitor 2A
ceRNAs: competing endogenous RNAs
CRISPR/Cas9: clustered regularly interspaced short palindromic repeats/CRISPR associate protein 9
cMRI: conventional magnetic resonance imaging
CTLA-4: cytotoxic T-lymphocyte-associated protein 4
DR4: Death receptor 4
DWI: diffusion weighted imaging
EGFR: epidermal growth factor receptor
EMR3: EGF module-containing, mucin-like hormone receptor 3
Emx2: empty spiracles homeobox 2
ER: endoplasmic reticulum
ESCs: embryo stem cells
FGFR3: fibroblast growth factor receptor 3
GBM: Glioblastoma
GPCRs: G protein-coupled receptors
GRK5: G protein-coupled receptor kinase 5
GRP78: glucose-regulated protein 78 kDa
GSC: glioblastoma stem-like cell
HGF/MET: hepatocyte growth factor / the mesenchymal-epithelial transition
HTI: hereditary tyrosinemia type I
HuR: Hu antigen R
IDH1: isocitrate dehydrogenase 1
IFNβ: interferon beta
IGF1R: insulin-like growth factor 1 receptor
IL-3: interleukin 3
iNSCs: induced neural stem cells
iPSCs: induced pluripotent stem cells
IQGAP1: IQ-domain GTPase-activating protein 1
lincRNAs: long intergenic non-coding RNAs
LTBP4: latent TGF-β-binding protein 4
LZTR1: leucine-zipper-like transcriptional regulator 1
MGMT: O^6^-methylguanine DNA methyltransferase
miRNAs: microRNAs
miRs: microRNAs
MNK1: MAP kinase-interacting kinase 1
MSH6: MutS homolog 6
NFκB: nuclear factor kappa B
NF1: neurofibromin 1
NSCs: tumor-tropic neural stem cells
OCTs: Octamer binding transcription factors
PDGFRA: platelet-derived growth factor receptor α polypeptide
PD-L1: programmed death-ligand 1
PIK3CA: phosphoinositide-3-kinase catalytic alpha
PIK3R1: phosphoinositide-3-kinase regulatory 1
PRDM2: PR domain containing 2
PTEN: phosphatase and tensin homolog
PTPN11: tyrosine-protein phosphatase non-receptor type 11
PWI: perfusion weighted imaging
P4HB: prolyl 4-hydroxylase beta polypeptide
RB1: retinoblastoma 1
SHH: sonic hedgehog homolog
S100A4/EMT: the S100 calcium-binding protein A4/the epithelial-mesenchymal transition
sgRNA: “single-guide” RNA
TACC3: transforming acidic coiled-coil containing protein 3
TCGA: the cancer genome atlas
TD: transdifferentiation
TERT: telomerase reverse transcriptase
TGFβ: transforming growth factor beta
TMZ: temozolomide
TP53: tumor protein p53
TRAIL: TNF-related apoptosis-inducing ligand
UPR: unfolded protein response
VEGF: vascular endothelial growth factor
WHO: world health organization

## References

[1] Verhaak RG, Hoadley KA, Purdom E, Wang V, Qi Y, Wilkerson MD, Miller CR, Ding L, Golub T, Mesirov JP, Alexe G, Lawrence M, O'Kelly M (2010). Integrated genomic analysis identifies clinically relevant subtypes of glioblastoma characterized by abnormalities in PDGFRA, IDH1, EGFR, and NF1. Cancer Cell.

[2] Brennan CW, Verhaak RG, McKenna A, Campos B, Noushmehr H, Salama SR, Zheng S, Chakravarty D, Sanborn JZ, Berman SH, Beroukhim R, Bernard B, Wu CJ (2013). The somatic genomic landscape of glioblastoma. Cell.

[3] Comprehensive genomic characterization defines human glioblastoma genes and core pathways (2008). Nature.

[4] Gebler C, Lohoff T, Paszkowski-Rogacz M, Mircetic J, Chakraborty D, Camgoz A, Hamann MV, Theis M, Thiede C, Buchholz F (2016). Inactivation of cancer mutations utilizing CRISPR/Cas9. J Natl Cancer Inst.

[5] Khan FA, Pandupuspitasari NS, Chun-Jie H, Ao Z, Jamal M, Zohaib A, Hakim MR, ShuJun Z (2016). CRISPR/Cas9 therapeutics: a cure for cancer and other genetic diseases. Oncotarget.

[6] Wang J, Cazzato E, Ladewig E, Frattini V, Rosenbloom DI, Zairis S, Abate F, Liu Z, Elliott O, Shin YJ, Lee JK, Lee IH, Park WY (2016). Clonal evolution of glioblastoma under therapy. Nat Genet.

[7] Frattini V, Trifonov V, Chan JM, Castano A, Lia M, Abate F, Keir ST, Ji AX, Zoppoli P, Niola F, Danussi C, Dolgalev I, Porrati P (2013). The integrated landscape of driver genomic alterations in glioblastoma. Nat Genet.

[8] Yan H, Parsons DW, Jin G, McLendon R, Rasheed BA, Yuan W, Kos I, Batinic-Haberle I, Jones S, Riggins GJ, Friedman H, Friedman A, Reardon D (2009). IDH1 and IDH2 mutations in gliomas. N Engl J Med.

[9] Feinberg AP Tycko B (2004). The history of cancer epigenetics. Nat Rev Cancer.

[10] Portela A, Esteller M (2010). Epigenetic modifications and human disease. Nat Biotechnol.

[11] Mack SC, Hubert CG, Miller TE, Taylor MD, Rich JN (2016). An epigenetic gateway to brain tumor cell identity. Nat Neurosci.

[12] Maleszewska M, Kaminska B (2013). Is glioblastoma an epigenetic malignancy?. Cancers (Basel).

[13] Williams MJ, Singleton WGB, Lowis SP, Malik K, Kurian KM (2017). Therapeutic targeting of histone modifications in adult and pediatric high-grade glioma. Front Oncol.

[14] Masui K, Cavenee WK, Mischel PS (2016). Cancer metabolism as a central driving force of glioma pathogenesis. Brain Tumor Pathol.

[15] Hegi ME, Diserens AC, Gorlia T, Hamou MF, de Tribolet N, Weller M, Kros JM, Hainfellner JA, Mason W, Mariani L, Bromberg JE, Hau P, Mirimanoff RO (2005). MGMT gene silencing and benefit from temozolomide in glioblastoma. N Engl J Med.

[16] Bell RJ, Rube HT, Kreig A, Mancini A, Fouse SD, Nagarajan RP, Choi S, Hong C, He D, Pekmezci M, Wiencke JK, Wrensch MR, Chang SM (2015). Cancer. The transcription factor GABP selectively binds and activates the mutant TERT promoter in cancer. Science.

[17] Killela PJ, Reitman ZJ, Jiao Y, Bettegowda C, Agrawal N, Diaz LA, Friedman AH, Friedman H, Gallia GL, Giovanella BC, Grollman AP, He TC, He Y (2013). TERT promoter mutations occur frequently in gliomas and a subset of tumors derived from cells with low rates of self-renewal. Proc Natl Acad Sci U S A.

[18] Shea A, Harish V, Afzal Z, Chijioke J, Kedir H, Dusmatova S, Roy A, Ramalinga M, Harris B, Blancato J, Verma M, Kumar D (2016). MicroRNAs in glioblastoma multiforme pathogenesis and therapeutics. Cancer Med.

[19] Mercatelli N, Galardi S, Ciafrè SA (2017). MicroRNAs as Multifaceted Players in Glioblastoma Multiforme. Int Rev Cell Mol Biol.

[20] Zhang Y, Xu Y, Li F, Li X, Feng L, Shi X, Wang L (2016). Dissecting dysfunctional crosstalk pathways regulated by miRNAs during glioma progression. Oncotarget.

[21] Shah N, Lankerovich M, Lee H, Yoon JG, Schroeder B, Foltz G (2013). Exploration of the gene fusion landscape of glioblastoma using transcriptome sequencing and copy number data. BMC Genomics.

[22] Costa R, Carneiro BA, Taxter T, Tavora FA, Kalyan A, Pai SA, Chae YK, Giles FJ (2016). FGFR3-TACC3 fusion in solid tumors: mini review. Oncotarget.

[23] Singh D, Chan JM, Zoppoli P, Niola F, Sullivan R, Castano A, Liu EM, Reichel J, Porrati P, Pellegatta S, Qiu K, Gao Z, Ceccarelli M (2012). Transforming fusions of FGFR and TACC genes in human glioblastoma. Science.

[24] Parker BC, Annala MJ, Cogdell DE, Granberg KJ, Sun Y, Ji P, Li X, Gumin J, Zheng H, Hu L, Yli-Harja O, Haapasalo H, Visakorpi T (2013). The tumorigenic FGFR3-TACC3 gene fusion escapes miR-99a regulation in glioblastoma. J Clin Invest.

[25] Nelson KN, Meyer AN, Siari A, Campos AR, Motamedchaboki K, Donoghue DJ (2016). Oncogenic gene fusion FGFR3-TACC3 is regulated by tyrosine phosphorylation. Mol Cancer Res.

[26] Di Stefano AL, Fucci A, Frattini V, Labussiere M, Mokhtari K, Zoppoli P, Marie Y, Bruno A, Boisselier B, Giry M, Savatovsky J, Touat M, Belaid H (2015). Detection, characterization, and inhibition of FGFR-TACC fusions in IDH wild-type glioma. Clin Cancer Res.

[27] Melendez J, Grogg M, Zheng Y (2011). Signaling role of Cdc42 in regulating mammalian physiology. J. Biol Chem.

[28] Okura H, Golbourn BJ, Shahzad U, Agnihotri S, Sabha N, Krieger JR, Figueiredo CA, Chalil A, Landon-Brace N, Riemenschneider A, Arai H, Smith CA, Xu S (2016). A role for activated Cdc42 in glioblastoma multiforme invasion. Oncotarget.

[29] Stacey M, Lin HH, Hilyard KL, Gordon S, McKnight AJ (2001). Human epidermal growth factor (EGF) module-containing mucin-like hormone receptor 3 is a new member of the EGF-TM7 family that recognizes a ligand on human macrophages and activated neutrophils. J Biol Chem.

[30] Kane AJ, Sughrue ME, Rutkowski MJ, Phillips JJ, Parsa AT (2010). EMR-3: a potential mediator of invasive phenotypic variation in glioblastoma and novel therapeutic target. Neuroreport.

[31] Kaur G, Kim J, Kaur R, Tan I, Bloch O, Sun MZ, Safaee M, Oh MC, Sughrue M, Phillips J, Parsa AT (2013). G-protein coupled receptor kinase (GRK)-5 regulates proliferation of glioblastoma-derived stem cells. J Clin Neurosci.

[32] Rooj AK, Bronisz A, Godlewski J (2016). The role of octamer binding transcription factors in glioblastoma multiforme. Biochim Biophys Acta.

[33] Kenific CM, Wittmann T, Debnath J (2016). Autophagy in adhesion and migration. J Cell Sci.

[34] Nakada M, Kita D, Watanabe T, Hayashi Y, Teng L, Pyko IV, Hamada J (2011). Aberrant signaling pathways in glioma. Cancers (Basel).

[35] Tuncbag N, Milani P, Pokorny JL, Johnson H, Sio TT, Dalin S, Iyekegbe DO, White FM, Sarkaria JN, Fraenkel E (2016). Network Modeling Identifies Patient-specific Pathways in Glioblastoma. Sci Rep.

[36] Kathagen-Buhmann A, Schulte A, Weller J, Holz M, Herold-Mende C, Glass R, Lamszus K (2016). Glycolysis and the pentose phosphate pathway are differentially associated with the dichotomous regulation of glioblastoma cell migration versus proliferation. Neuro Oncol.

[37] Seyfried TN, Flores R, Poff AM, D'Agostino DP, Mukherjee P (2015). Metabolic therapy: a new paradigm for managing malignant brain cancer. Cancer Lett.

[38] Li X, Wu C, Chen N, Gu H, Yen A, Cao L, Wang E, Wang L (2016). PI3K/Akt/mTOR signaling pathway and targeted therapy for glioblastoma. Oncotarget.

[39] Sami A, Karsy M (2013). Targeting the PI3K/AKT/mTOR signaling pathway in glioblastoma: novel therapeutic agents and advances in understanding. Tumour Biol.

[40] Holmen SL, Williams BO (2005). Essential role for Ras signaling in glioblastoma maintenance. Cancer Res.

[41] Robinson JP, Vanbrocklin MW, McKinney AJ, Gach HM, Holmen SL (2011). Akt signaling is required for glioblastoma maintenance *in vivo*. Am J Cancer Res.

[42] Mason WP, Belanger K, Nicholas G, Vallieres I, Mathieu D, Kavan P, Desjardins A, Omuro A, Reymond D (2012). A phase II study of the Ras-MAPK signaling pathway inhibitor TLN-4601 in patients with glioblastoma at first progression. J Neurooncol.

[43] Logan CY, Nusse R (2004). The Wnt signaling pathway in development and disease. Annu Rev Cell Dev Biol.

[44] Clevers H, Nusse R (2012). Wnt/beta-catenin signaling and disease. Cell.

[45] Lee Y, Lee JK, Ahn SH, Lee J, Nam DH (2016). WNT signaling in glioblastoma and therapeutic opportunities. Lab Invest.

[46] Yang W, Yu H, Shen Y, Liu Y, Yang Z, Sun T (2016). MiR-146b-5p overexpression attenuates stemness and radioresistance of glioma stem cells by targeting HuR/lincRNA-p21/beta-catenin pathway. Oncotarget.

[47] Kaminska B, Kocyk M, Kijewska M (2013). TGF beta signaling and its role in glioma pathogenesis. Adv Exp Med Biol.

[48] Joseph JV, Balasubramaniyan V, Walenkamp A, Kruyt FA (2013). TGF-beta as a therapeutic target in high grade gliomas - promises and challenges. Biochem Pharmacol.

[49] Massague J (2008). TGFbeta in cancer. Cell.

[50] Han J, Alvarez-Breckenridge CA, Wang QE, Yu J (2015). TGF-beta signaling and its targeting for glioma treatment. Am J Cancer Res.

[51] Grzmil M, Morin P, Lino MM, Merlo A, Frank S, Wang Y, Moncayo G, Hemmings BA (2011). MAP kinase-interacting kinase 1 regulates SMAD2-dependent TGF-beta signaling pathway in human glioblastoma. Cancer Res.

[52] Le Reste PJ, Avril T, Quillien V, Morandi X, Chevet E (2016). Signaling the unfolded protein response in primary brain cancers. Brain Res.

[53] Penaranda Fajardo NM, Meijer C, Kruyt FA (2016). The endoplasmic reticulum stress/unfolded protein response in gliomagenesis, tumor progression and as a therapeutic target in glioblastoma. Biochem Pharmacol.

[54] Pyrko P, Schonthal AH, Hofman FM, Chen TC, Lee AS (2007). The unfolded protein response regulator GRP78/BiP as a novel target for increasing chemosensitivity in malignant gliomas. Cancer Res.

[55] Epple LM, Dodd RD, Merz AL, Dechkovskaia AM, Herring M, Winston BA, Lencioni AM, Russell RL, Madsen H, Nega M, Dusto NL, White J, Bigner DD (2013). Induction of the unfolded protein response drives enhanced metabolism and chemoresistance in glioma cells. PLoS One.

[56] Yoo RE, Choi SH (2016). Recent Application of Advanced MR Imaging to Predict Pseudoprogression in High-grade Glioma Patients. Magn Reson Med Sci.

[57] Hyare H, Thust S, Rees J (2017). Advanced MRI Techniques in the Monitoring of Treatment of Gliomas. Curr Treat Options Neurol.

[58] Aquino D, Gioppo A, Finocchiaro G, Bruzzone MG, Cuccarini V (2017). MRI in Glioma Immunotherapy: Evidence, Pitfalls, and Perspectives. J Immunol Res.

[59] Stupp R, Hegi ME, Mason WP, van den Bent MJ, Taphoorn MJ, Janzer RC, Ludwin SK, Allgeier A, Fisher B, Belanger K, Hau P, Brandes AA, Gijtenbeek J (2009). Effects of radiotherapy with concomitant and adjuvant temozolomide versus radiotherapy alone on survival in glioblastoma in a randomised phase III study: 5-year analysis of the EORTC-NCIC trial. Lancet Oncol.

[60] Stupp R, Mason WP, van den Bent MJ, Weller M, Fisher B, Taphoorn MJ, Belanger K, Brandes AA, Marosi C, Bogdahn U, Curschmann J, Janzer RC, Ludwin SK (2005). Radiotherapy plus concomitant and adjuvant temozolomide for glioblastoma. N Engl J Med.

[61] Stummer W, Pichlmeier U, Meinel T, Wiestler OD, Zanella F, Reulen HJ (2006). Fluorescence-guided surgery with 5-aminolevulinic acid for resection of malignant glioma: a randomised controlled multicentre phase III trial. Lancet Oncol.

[62] Jue TR, McDonald KL (2016). The challenges associated with molecular targeted therapies for glioblastoma. J Neurooncol.

[63] Crespo I, Vital AL, Gonzalez-Tablas M, Patino Mdel C, Otero A, Lopes MC, de Oliveira C, Domingues P, Orfao A, Tabernero MD (2015). Molecular and genomic alterations in glioblastoma multiforme. Am J Pathol.

[64] Prados MD, Byron SA, Tran NL, Phillips JJ, Molinaro AM, Ligon KL, Wen PY, Kuhn JG, Mellinghoff IK, de Groot JF, Colman H, Cloughesy TF, Chang SM (2015). Toward precision medicine in glioblastoma: the promise and the challenges. Neuro Oncol.

[65] van Tellingen O, Yetkin-Arik B, de Gooijer MC, Wesseling P, Wurdinger T, de Vries HE (2015). Overcoming the blood-brain tumor barrier for effective glioblastoma treatment. Drug Resist Updat.

[66] Eyler CE, Rich JN (2008). Survival of the fittest: cancer stem cells in therapeutic resistance and angiogenesis. J Clin Oncol.

[67] Chen J, Li Y, Yu TS, McKay RM, Burns DK, Kernie SG, Parada LF (2012). A restricted cell population propagates glioblastoma growth after chemotherapy. Nature.

[68] Chen R, Nishimura MC, Bumbaca SM, Kharbanda S, Forrest WF, Kasman IM, Greve JM, Soriano RH, Gilmour LL, Rivers CS, Modrusan Z, Nacu S, Guerrero S (2010). A hierarchy of self-renewing tumor-initiating cell types in glioblastoma. Cancer Cell.

[69] Karim R, Palazzo C, Evrard B, Piel G (2016). Nanocarriers for the treatment of glioblastoma multiforme: Current state-of-the-art. J Control Release.

[70] Fakhoury M (2016). Drug delivery approaches for the treatment of glioblastoma multiforme. Artif Cells Nanomed Biotechnol.

[71] Theeler BJ, Gilbert MR (2015). Advances in the treatment of newly diagnosed glioblastoma. BMC Med.

[72] Qazi MA, Vora P, Venugopal C, Sidhu SS, Moffat J, Swanton C, Singh SK (2017). Intratumoral heterogeneity: pathways to treatment resistance and relapse in human glioblastoma. Ann Oncol.

[73] Kane JR, Miska J, Young JS, Kanojia D, Kim JW, Lesniak MS (2015). Sui generis: gene therapy and delivery systems for the treatment of glioblastoma. Neuro Oncol.

[74] Falcone C, Daga A, Leanza G, Mallamaci A (2016). Emx2 as a novel tool to suppress glioblastoma. Oncotarget.

[75] Wollmann G, Ozduman K, van den Pol AN (2012). Oncolytic virus therapy for glioblastoma multiforme: concepts and candidates. Cancer J.

[76] Desjardins A, Sampson JH, Peters KB, Vlahovic G, Randazzo D, Threatt S, Herndon JE, Boulton S, Lally-Goss D, McSherry F, Lipp ES, Friedman AH, Friedmen HS (2015). Oncolytic polio/rhinovirus recombinant (PVSRIPO) against recurrent glioblastoma (GBM): Optimal dose determination. J Clin Oncol.

[77] Goetz C, Dobrikova E, Shveygert M, Dobrikov M, Gromeier M (2011). Oncolytic poliovirus against malignant glioma. Future Virol.

[78] Brown MC, Gromeier M (2015). Cytotoxic and immunogenic mechanisms of recombinant oncolytic poliovirus. Curr Opin Virol.

[79] Denniston E, Cresdon H, Rucinsky N, Stegman A, Remenar D, Moio K, Clark B, Higginbotham A, Keffer R, Brammer S, Horzempa J (2016). The practical consideration of poliovirus as an oncolytic virotherapy. AM J Virol.

[80] Fueyo J, Alemany R, Gomez-Manzano C, Fuller GN, Khan A, Conrad CA, Liu TJ, Jiang H, Lemoine MG, Suzuki K, Sawaya R, Curiel DT, Yung WK (2003). Preclinical characterization of the antiglioma activity of a tropism-enhanced adenovirus targeted to the retinoblastoma pathway. J Natl Cancer Inst.

[81] Jiang H, Clise-Dwyer K, Ruisaard KE, Fan X, Tian W, Gumin J, Lamfers ML, Kleijn A, Lang FF, Yung WK, Vence LM, Gomez-Manzano C, Fueyo J (2014). Delta-24-RGD oncolytic adenovirus elicits anti-glioma immunity in an immunocompetent mouse model. PLoS One.

[82] Lang FF, Conrad C, Gomez-Manzano C, Tufaro F, Yung WKA, Sawaya R, Weinberg J, Prabhu S, Fuller G, Aldape K, Fueyo J (2014). First-in-human phase I clinical trial of oncolytic Delta-24-RGD (DNX-2401) with biological endpoints: implications for viro-immunotherapy. Neuro Oncol.

[83] Jiang H, Rivera-Molina Y, Gomez-Manzano C, Clise-Dwyer K, Bover L, Vence LM, Yuan Y, Lang FF, Toniatti C, Hossain MB, Fueyo J (2017). Oncolytic adenovirus and tumor-targeting immune modulatory therapy improve autologous cancer vaccination. Cancer Res.

[84] Gruslova A, Cavazos DA, Miller JR, Breitbart E, Cohen YC, Bangio L, Yakov N, Soundararajan A, Floyd JR, Brenner AJ (2015). VB-111: a novel anti-vascular therapeutic for glioblastoma multiforme. J Neurooncol.

[85] GuhaSarkar D, Su Q, Gao G, Sena-Esteves M (2016). Systemic AAV9-IFNbeta gene delivery treats highly invasive glioblastoma. Neuro Oncol.

[86] Yang I, Han SJ, Sughrue ME, Tihan T, Parsa AT (2011). Immune cell infiltrate differences in pilocytic astrocytoma and glioblastoma: evidence of distinct immunological microenvironments that reflect tumor biology. J Neurosurg.

[87] Yang I, Tihan T, Han SJ, Wrensch MR, Wiencke J, Sughrue ME, Parsa AT (2010). CD8+ T-cell infiltrate in newly diagnosed glioblastoma is associated with long-term survival. J Clin Neurosci.

[88] Razavi SM, Lee KE, Jin BE, Aujla PS, Gholamin S, Li G (2016). Immune evasion strategies of glioblastoma. Front Surg.

[89] Finocchiaro G, Pellegatta S (2015). Novel mechanisms and approaches in immunotherapy for brain tumors. Discov Med.

[90] Dunn-Pirio AM, Viahovic G (2017). Immunotherapy approaches in the treatment of malignant brain tumors. Cancer.

[91] Johnson LA, Scholler J, Ohkuri T, Kosaka A, Patel PR, McGettigan SE, Nace AK, Dentchev T, Thekkat P, Loew A, Boesteanu AC, Cogdill AP, Chen T (2015). Rational development and characterization of humanized anti-EGFR variant III chimeric antigen receptor T cells for glioblastoma. Sci Transl Med.

[92] Uzzaman M, Keller G, Germano IM (2009). *In vivo* gene delivery by embryonic-stem-cell-derived astrocytes for malignant gliomas. Neuro Oncol.

[93] Bago JR, Alfonso-Pecchio A, Okolie O, Dumitru R, Rinkenbaugh A, Baldwin AS, Miller CR, Magness ST, Hingtgen SD (2016). Therapeutically engineered induced neural stem cells are tumour-homing and inhibit progression of glioblastoma. Nat Commun.

[94] Guemet A, Grumolato L (2017). CRISPR/Cas9 editing of the genome for cancer modeling. Methods.

[95] Luo J (2016). CRISPR/Cas9: from genome engineering to cancer drug discovery. Trends Cancer.

[96] Guernet A, Mungamuri SK, Cartier D, Sachidanandam R, Jayaprakash A, Adriouch S, Vezain M, Charbonnier F, Rohkin G, Coutant S, Yao S, Ainani H, Alexandre D (2016). CRISPR-barcoding for intratumor genetic heterogeneity modeling and functional analysis of oncogenic driver mutations. Mol Cell.

[97] Yang W, Tu Z, Sun Q, Li XJ (2016). CRISPR/Cas9: Implications for modeling and therapy of neurodegenerative diseases. Front Mol Neurosci.

[98] Zuckermann M, Hovestadt V, Knobbe-Thomsen CB, Zapatka M, Northcott PA, Schramm K, Belic J, Jones DT, Tschida B, Moriarity B, Largaespada D, Roussel MF, Korshunov A (2015). Somatic CRISPR/Cas9-mediated tumour suppressor disruption enables versatile brain tumour modelling. Nat Commun.

[99] Wu WH, Tsai YT, Justus S, Lee TT, Zhang L, Lin CS, Bassuk AG, Mahajan VB, Tsang SH (2016). CRISPR repair reveals causative mutation in a preclinical model of retinitis pigmentosa. Mol Ther.

[100] Yin H, Xue W, Chen S, Bogorad RL, Benedetti E, Grompe M, Koteliansky V, Sharp PA, Jacks T, Anderson DG (2014). Genome editing with Cas9 in adult mice corrects a disease mutation and phenotype. Nat Biotechnol.

[101] Xue HY, Zhang X, Wang Y, Xiaojie L, Dai WJ, Xu Y (2016). *In vivo* gene therapy potentials of CRISPR-Cas9. Gene Ther.

